# Quantifying the Core Deficit in Classical Schizophrenia From Three Independent Samples of Psychosis Spectrum Patients

**DOI:** 10.1192/bjo.2024.74

**Published:** 2024-08-01

**Authors:** Mohanbabu Rathnaiah, Elizabeth Liddle, Peter Liddle

**Affiliations:** 1 Derbyshire Healthcare NHS Foundation Trust, Derby, United Kingdom; 2 University of Nottingham, Nottingham, United Kingdom

## Abstract

**Aims:**

In schizophrenia, disorganisation and impoverished mental activity (as described in the classical descriptions by Kraepelin and Bleuler) together with impaired cognitive function, predict persisting functional impairment (Liddle, 2019). We propose that in schizophrenia, four ‘classical’ features (disorganisation, impoverished mental activity, cognitive dysfunction, and impaired role-functioning) arise from a shared pathophysiological process that increases risk of persisting functional impairment. We also propose that this shared process creates a risk of subsequent episodic reality distortion (delusions and hallucinations). In the current work, we investigate whether a single latent variable accounts for the shared variance in the four ‘classical’ features. We also investigate whether the severity of this latent variable based on assessment of long-standing classical symptoms predicts severity of current reality distortion.

**Methods:**

We performed maximum likelihood factor analysis of disorganisation, impoverishment, cognition and role-function in three separate samples of patients (n = 54, n = 128, n = 64) with DSM diagnosed schizophrenia, schizo-affective disorder or bipolar disorder. In the first two samples, we quantified current disorganisation and impoverished mental activity using the Positive and Negative Syndrome Scale (PANSS). In the third, we scored persistent disorganisation and impoverished mental activity according to Symptoms & Signs of Psychosis Illness (SSPI) based on systematic examination of case records. We assessed cognition using the Digit Symbol Substitution Test (DSST) and role-function using the Social & Occupational functioning scale (in two studies) and the Personal & Social Performance scale (in one study). We quantified current reality distortion by summing SSPI scores for current delusions and hallucinations.

**Results:**

In each of the three studies, a single latent variable accounted for more than 50% of variance. Loadings were similar whether current or persistent symptoms were used. The latent variable derived from persistent symptom scores correlated significantly with current reality distortion.

**Conclusion:**

This series of studies provide further evidence that disorganisation, impoverished mental activity, cognitive impairment and impaired role function share substantial variance, consistent with the proposal that they reflect a core pathophysiological process underlying ‘classical’ schizophrenia. Furthermore, our findings are consistent with the hypothesis that over time, this pathophysiological process increases the risk of episodic reality distortion. However, these were all cross-sectional studies, and need to be confirmed using longitudinal data. Our findings have potential clinical and research implications including development of a custom-made clinical tool to quantify the core deficit as well as investigating targeted interventions employing medication or neuromodulation.

